# HLA Genes in Mayos Population from Northeast Mexico

**DOI:** 10.2174/138920207783591735

**Published:** 2007-11

**Authors:** A Arnaiz-Villena, J Moscoso, J Granados, J.I Serrano-Vela, A. de la Peña, R Reguera, A Ferri, E Seclen, R Izaguirre, N Perez-Hernandez, G Vargas-Alarcon

**Affiliations:** 1 Department of Immunology, Universidad Complutense, The Madrid Regional Blood Center, Madrid, Spain;; 2 Department of Immunology and Rheumatology, Instituto Nacional de Ciencias Medicas y Nutricion Salvador Zubiran, Mexico City, Mexico; 3 Department of Physiology and Grupo de Estudio de Genomica y Proteomica en Enfermedades Cardiovasculares, Instituto Nacional de Cardiologia Ignacio Chavez, Mexico City, Mexico

**Keywords:** Amerindians, HLA, mayos, mexica, nahua, transplant.

## Abstract

HLA class I and class II alleles have been studied in 60 unrelated people belonging to Mayos ethnic group, which lives in the Mexican Pacific Sinaloa State. Mayos HLA profile was compared to other Amerindians and worldwide populations’ profile. A total of 14,896 chromosomes were used for comparisons. Genetic distances between populations, Neigbour-Joining dendrograms and correspondence analyses were performed to determine the genetic relationship among population. The new specific Mayo HLA haplotypes found are: HLA-A*02-B*35-DRB1*1406-DQB1*0301; HLA-A*02-B*48-DRB1*0404-DQB1*0302; HLA-A*24-B*51-DRB1*0407-DQB1*0302 and HLA-A*02-B*08-DRB1*0407-DQB1*0302. However, the typical Meso American HLADRB1*0407 represents a 40% of all DRB1 alleles. While common HLA characteristics are found in Amerindian distant ethnic groups, still new group specific HLA haplotypes are being found, suggesting that a common founder effect (i.e. high DRB1*0407) is noticed. Moreover, new HLA haplotypes are almost certainly appearing along time probably due to specific pathogen (?) selection for diversity. Mayo language is close to the Tarahumara one (another geographically close group); notwithstanding both groups are not genetically close according to our results, showing again the different evolution of genes and languages, which do not correlate. Finally, Sinaloa is one of the Mexican States in which more European genes are found. However, the results presented in this paper, where no European HLA genes are seen in Mayos, should have a bearing in establishing transplant programs and in HLA and disease studies.

## INTRODUCTION

The West Mexican Mayos form an isolated ethnic Amerindian group, which lives on a flat half desert and half cultivated area, Southern Sonora and North Sinaloa, see Fig. (**1**). They speak a Nahua/Aztec type language with some dialects that do not hinder mutual understanding. They call themselves Yoremes and probably migrated to the present day location from the North (Colorado River Basin) about 1,000 years ago [1]. They usually get married within their own group [1] which is important for the analyses of our results. Whether the Nahua type language (Mayo) also a sign of Mexica or Aztec relatedness is unknown, since Mexicas imposed their language to other probably non-genetically related Mexican groups [2]. Later, the Spanish conqueror Hernan Cortes arrived to the Mexicas Empire in 1,519 AD which was sized and destroyed by war and invaders’ born-diseases [3]. 

The present study aims: 1) To relate Mayos group with other Amerindian groups; and 2) To relate Mayos with other worldwide groups, in order to test the uniqueness of all Amerindians [4] with respect to their HLA profiles (different loci allele frequencies). This is important for establishing transplant programs in rural areas where some ethnic groups still live, i.e. Mayos, and for studying the epidemiology of HLA linked disorders.

In order to perform this work, a sample of sixty unrelated Mayos has been HLA typed and thereafter the genetic relationship with others Amerindians tested by HLA-A, -B, -DRB1 and –DQB1 DNA typing calculating genetic distances, neighbor-joining dendrograms and correspondence analyses.

## MATERIALS AND METHODS

### Population Samples

We studied sixty unrelated healthy individuals from the Mayos ethnic group. They were HLA class I and class II typed. They were living at Capomos town, Municipio del Fuerte, in Sinaloa State, northeast Mexico. In the Mayos communities the inbreeding pedigrees are common but we selected only those unrelated individuals for our study. Each individual was born in the Mayos area, their four grandparents had been born in the same area and spoke their own Mayo language. All participants gave their written consent for being included in the present study.

The origin of all other populations used for comparisons is detailed in Table **1**: 14,896 chromosomes were studied in total, including populations from different origins (Caucasoids, Orientals, Polynesians, Micronesians, Na-Dene, Eskimos and Amerindians). In particular, the Amerindian group includes tribes from the following linguistic families: Macro-Mixteco (Mixtecans, Zapotecans and Mazatecans), Macro-Maya (Mixe and Mayas), Macro-Yuma (Seris), Andean (Quechuans and Aymaras), Chibcha (Arsario, Kogi, Arhuaco and Cayapa), Arawak (Wayu), and Ge Pano Caribe (Xavantes, Mataco and Toba).

### HLA Typing and DNA Sequencing

HLA class I (A and B) and class II (DRB1 and DQB1) allele families typing was performed by polymerase chain reaction-sequence specific oligonucleotide (PCR-SSO) reverse dot blot hybridization (Amplicor, Hoffmann La Roche, Basel, Switzerland) (according to IMGT/HLA version 2.6, July 2004). HLA-DRB1 and DQB1 high-resolution typing was done by DNA sequencing in a Perkin Elmer 310 automated DNA sequencer (Foster City, CA, USA). The sequences were analyzed using the IMGT/HLA sequence database (http://www.ebi.ac.uk/imgt/hla/align.html).

### Statistical Analysis

Statistical analysis was performed with Arlequin v2.0 software kindly provided by Schneider [5]. In summary, this program calculated HLA-A, -B, -DRB1, and –DQB1 allele frequencies, Hardy-Weinberg equilibrium and the linkage disequilibrium (D’; also named LD) [6] and their level of significance (P) for 2X2 comparisons was determined [7] and the 11^th^ International Histocompatibility Workshop methodology [6]. In addition, the most frequent complete haplotypes were deduced from: (1) the 2, 3, and 4 HLA loci haplotype frequencies [8]; (2) the previously described haplotypes in other populations [9]; and (3) haplotypes if they appeared in two or more individuals and if the alternative haplotype was well defined [9]. In order to compare phenotype and haplotype HLA frequencies with other populations, the reference tables of the 11^th^ and 12^th^ International HLA Workshops are used (see also Table **1**) [10, 11]. Dendograms were constructed with the allelic frequencies using the neighbor-joining (NJ) method [12] with the genetic distances (DA) between populations [13], using DISPAN software comprising the programs GNKDST and TREEVIEW [14, 15]. Correspondence analysis in n-dimensions and its bi-dimensional representation was carried out using the VISTA v5.02 computer program (http://www.forrest.psych.unc.edu) [16]. Correspondence analysis consists of a geometric technique that may be used for displaying a global view of the relationships among populations according to HLA (or other) allele frequencies. This methodology is based on the allelic frequency variance among populations and on the display of a statistical visualization of differences.

## RESULTS

### Characteristic HLA Allele Frequencies in the Mayos Population Compared to other Populations

The expected and observed genotype frequency values for HLA-A, -B, and –DQB1 loci were in Hardy-Weinberg equilibrium. Table **2** shows the HLA frequencies found in the Mayos population. HLA-A and –B were typed on 60 individuals. Ten different HLA-A and fifteen different HLA-B alleles were found in the Mayos ethnic group. The most frequent HLA-A alleles were A*02 (allele frequency=0.44) and A*24 (allele frequency=0.36) whereas the most frequent HLA-B alleles were B*35 (allele frequency=0.25), B*39 (allele frequency=0.17) and B*51 (allele frequency= 0.17).

With respect to the HLA class II, 60 individuals were typed. Eighteen different HLA-DRB1 alleles were found, but only two of them had frequencies higher than 0’1 (DRB1*0407 and DRB1*1406), the sum of these two allelic frequencies was more than 0’6. Other three alleles common in Amerindians have been found in this ethnic group with allelic frequencies between 0’05-0’1 (DRB1*0403, DRB1*0404 and DRB1*0802). DQB1 allele frequencies reflect the DRB1 locus allele distribution due to the strong linkage disequilibrium level between these two loci, thus, the most frequent HLA-DQB1 alleles were DQB1*0302 (allele frequency=0.63), DQB1*0301 (allele frequency=0.18) and DQB1*0402 (allele frequency=0.11).

Two types of analysis were done in order to compare Mayos HLA frequencies with other world population’s frequencies: 1) with pooled DRB1 and DQB1 data; and 2) with DRB1 only. It was not possible to carry out a study comparing frequencies of HLA class I and II conjointly due to the lack of class I studies in many Amerindian and other populations. The single DRB1 study was carried out in order to compare the American Indian HLA population frequencies with those of Polynesians, Melanesians and Micronesians, who lacked DQB1 analyses, see Table **1**, Table **3** and Fig. (**2**). HLA-DRB1, DQB1 neighbor-joining tree grouped together the Amerindians and separated them from Na-Dene and Eskimo Native American groups and also from the Orientals and Caucasoids, Fig. (**3**). When the Polynesians, Melanesians and Micronesians were included (see Table **1**) the topology of the tree (DRB1 alone) does not change (data not shown), it is also seen in the correspondence analysis, Fig. (**2**). This effect is more evident when genetic distances are considered (Table **3**).

Genetic distances between Mayos and other populations show that Mayos are both close to Meso and South Americans (Table **3**). Indeed, Kogi (11.20x10^-2^, Colombia), Teenek (11.38x10^-2^, Mexico), Mayans (13.17 x10^-2^, Guatemala), Arsario (18.13 x10^-2^, Colombia), Mazatecans (20.44 x10^-2^, Mexico) and Mixteco (23.20 x10^-2^, Mexico) show the closest genetic distances to Mayos, followed by Eastern Toba, Arhuaco, Seri, Nahuas and the rest of Amerindian populations. A discontinuity between Amerindians and the other world groups (including Na-Dene and Eskimos) is observed. With respect to Guarani population, we could observe that a more larger genetic distance exists with Mayos; larger than with other non Amerindian population (Athabaskans), this fact may be due to the low number of HLA typings of the Guarani population or to admixture (Table **3**).

### HLA-A, -B, -DRB1 and –DQB1 Linkage Disequilibrium Analysis in Mayos Compared to other Populations

Associations between different HLA loci were estimated. The most probable two loci haplotype combinations (A-B, B-DRB1 and DRB1-DQB1) were calculated (data not shown).

The ten most frequent extended haplotypes are depicted in Table **4**. Mayos extended HLA haplotypes have been obtained for the first time, allowing their comparison with the previously reported ones in other populations (Table **4** and its footnote).

The DRB1*0407-DQB1*0302 was associated with A*24-B*39, A*02-B*35, A*24-B*35, A*02-B*40, A*24-B*51, A*02-B*39 and A*02-B*08, meanwhile DRB1*1406-DQB1*0301 was associated with A*2-B*35; DRB1*0404-DQB1*0302 was associated with A*02-B*48 and DRB1*0802-DQB1*0402 was associated with A*02-B*35.

The most frequent extended HLA haplotypes have Amerindian characteristics and it has been found in other Amerindian populations. The most frequent extended HLA haplotype A*24-B*39-DRB1*0407-DQB1*0302 (8.2%) has also been found only in Mazatecan Amerindian population. Six of the ten most frequent Mayos haplotypes have been found in other Amerindians populations, mainly in other Meso-American populations as Mazatecan, Seri, Mixe, Teeneks, Mixtecos, Mayans, Nahuas and Zapotecans. Four of them have not been found in any other worldwide ethnic group, these haplotypes seem to be specific for this Mexican ethnic group (Table **4** and its footnote).

## DISCUSSION

### Mayos and Mexican Populations

We studied an isolated population from Northeast Mexico (Mayos) which confirmed the limited HLA polymorphism found in other isolated tribes from Meso and South America. We detected only ten HLA-A alleles and fifteen HLA-B different alleles in this population. Only two (A*02 and A*24) HLA-A alleles and three (B*35, B*40 and B*51) HLA-B alleles had frequencies higher than 0’1. With respect to HLA class II genes, only two (DRB1*0407 and DRB1*1406) HLA-DRB1 alleles and three (DQB1*0302, DQB1*0301 and DQB1*0402) HLA-DQB1 alleles had frequencies higher than 0’1. All of these HLA class I and II alleles have already been described in other Mexican populations [2, 4, 17, 18, 19].

Neighbour-Joining dendrogram and correspondence analyses correlate Mayos tribe together with Mexican populations like Teeneks and Tarahumaras, Fig. (**3**), and also with Nahuas ethnic group, Fig. (**2**). Regarding the genetic distances (a bidimensional parameter), Kogi from Colombia, and Mayans from Guatemala are the populations most related to Mayos (Table **3**).

We can deduce from HLA genetic results (allele frequencies and extended haplotypes) that our Mayos sample has not had any contact with European people, because it would be clearly shown by using these particular HLA genetic markers [20].

### Mayos and South American Populations

The results of HLA genetic typing in Mayos population confirm that they belong to Amerindian HLA genetic profile.

The presence of HLA-B*48 allele in this population is remarkable (allele frequency= 0’05, Table **2**) as it is lacking in all Mexican populations so far except in Andean populations like Aymaras, Quechuas and Lamas [21-23].

HLA-B*48 is relatively common in Na-Dene and Asian populations [9, 24]. The presence of this allele (together with DRB1*0901) in a few Amerindian populations is the only evidence that could strictly support the three wave model of peopling America including Meso and South America [25] because these two alleles would be shared with southern Asian populations.

All Amerindian ethnic groups bearing HLA-B*48 allele in its HLA pofile are living in the Pacific Coast (Mayos and Andean Populations). This might mean the existence of southern Asian migrations to coastal Meso and South America.

### Amerindian Languages

Again, while Mayos seem to be genetically related with South American populations [Kogi, Table **1**; Terena and Aymaras, Fig. (**3**); and Wayu and Quechua, Fig. (**2**)], languages spoken by these groups are disparate and belong to far related linguistic branches.

It is also remarkable that Tarahumara ethnic group, which speaks a similar language to Mayos and is geographically very close to it, genetically shows relatively distant, Table **2**, Fig. (**2**) and Fig. (**3**). This is a general phenomenon in Amerindians: genes and languages do not correlate.

Finally, Mayos ethnic group lives in Sinaloa Mexican State and at least in our sample European HLA genes and haplotypes are not found; this occurs in spite of that Sinaloa is one of the Mexican States where more European HLA alleles are found [20].

These facts may be taken into account when setting up transplantation programs and also for studiyng HLA and disease associations.

## Figures and Tables

**Fig. (1):**
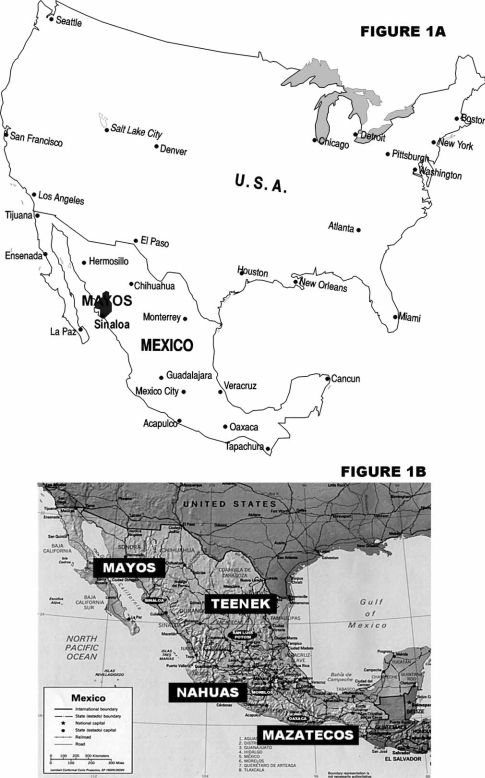
**A**) North American map showing Mayos living region in Mexican Sinaloa State in dark grey; **B**) Map of Mexico also showing different Mexican populations previously studied.

**Fig. (2):**
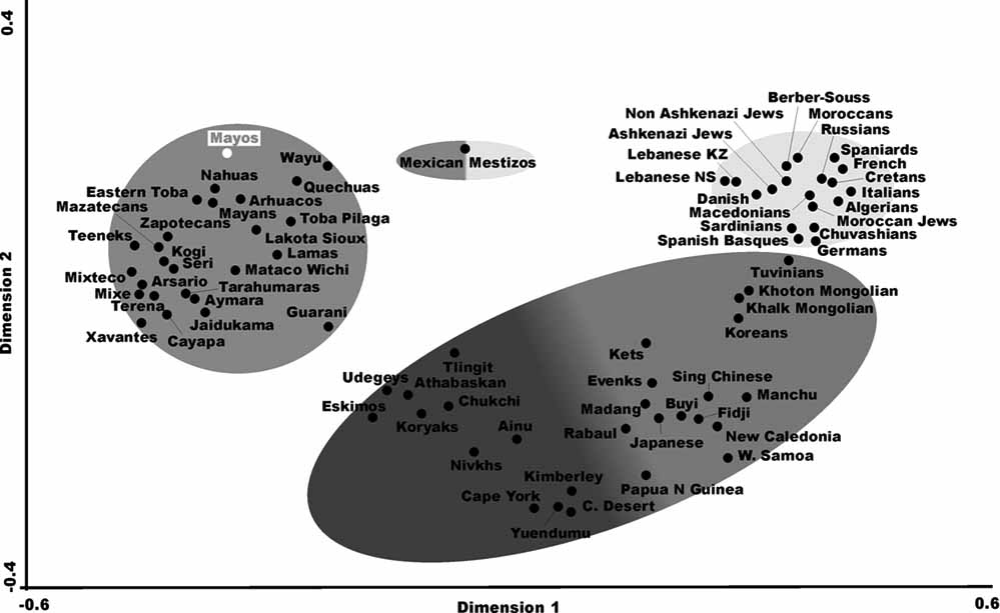
Correspondence analysis showing a global view of the relationship among Amerindian, Na-Dene, Eskimo, Asian, European and Polynesian populations according to HLA-DRB1 allele frequencies in n dimensions (bi-dimensional representation). Grey intensity represents an approximate grouping of populations.

**Fig. (3):**
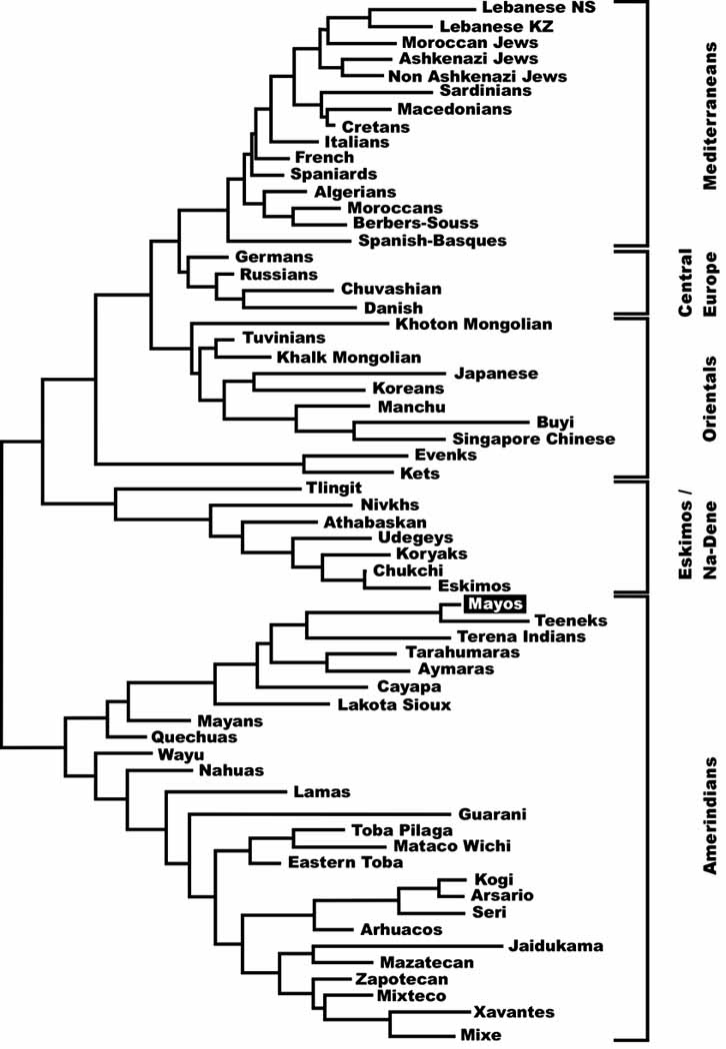
Neighbor-joining dendrogram showing relatedness between Mayos and Amerindians, Na-Dene, Eskimo, Asian and European populations. Genetic distances between populations (DA) were calculated by using HLA-DRB1-DQB1 genotyping. Data from other populations were taken from references detailed in Table **1**.

**Table 1:** Populations Included in the Analysis. A Total of 14,896 Chromosomes were Analyzed. Geographical Locations are Represented in [26]

ID	Populations	N	Ref.	ID	Populations	N	Ref.
1	Mayos	60	-	38	Buyi	70	[9]
2	Nahuas	85	[2]	39	Manchu	50	[33]
3	Aymaras	102	[21]	40	Koreans	100	[9]
4	Quechuans	80	[22]	41	Japanese	493	[9]
5	Mazatecans	89	[4]	42	Khalk Mongolians	202	[34]
6	Mayans	132	[26]	43	Tuvinians	197	[35]
7	Seri	100	[17]	44	Khoton Mongolians	85	[34]
8	Mixe	55	[17]	45	Germans	295	[9]
9	Mixtecans	103	[17]	46	Sardinians	91	[9]
10	Zapotecans	75	[17]	47	Italians	284	[9]
11	Mexican Mestizos	99	Unpublished	48	French	179	[9]
12	Wayu	112	[27]	49	Spaniards	176	[36]
13	Arhuaco	123	[27]	50	Spanish Basques	80	[36]
14	Kogi	67	[27]	51	Algerians	102	[8]
15	Arsario	20	[27]	52	Berbers (Souss)	98	[37]
16	Cayapa	100	[28]	53	Moroccans	98	[38]
17	Xavantes	74	[29]	54	Macedonians	172	[39]
18	Guarani	32	[17]	55	Cretans	135	[40]
19	Toba Pilaga	19	[29]	56	Ashkenazi Jews	80	[41]
20	Mataco Wichi	49	[29]	57	Non Ashkenazi Jews	80	[41]
21	Eastern Toba	135	[29]	58	Lebanese NS	59	[11]
22	Jaidukama	39	Unpublished	59	Lebanese KZ	93	[11]
23	Teenek	55	[19]	60	Moroccan Jews	94	[42]
24	Lakota Sioux	302	[30]	61	Danish	124	[9]
25	Terena indians	60	[31]	62	Chuvashians	82	[43]
26	Tarahumaras	44	[18]	63	Russians	200	[44]
27	Lamas	83	[23]	64	Western Samoa	102	[45]
28	Eskimos	35	[32]	65	Madang	65	[46]
29	Athabaskans	124	[24]	66	Rabaul	60	[46]
30	Tlingit	53	[9]	67	New Caledonia	65	[46]
31	Nivkhs	32	[32]	68	Fidji	57	[46]
32	Udegeys	23	[32]	69	Papua New Guinea	57	[46]
33	Koryaks	92	[32]	70	Central Desert	152	[47]
34	Chukchi	59	[32]	71	Ainu	50	[48]
35	Kets	22	[32]	72	Yuendumu	119	[47]
36	Evenks	35	[32]	73	Cape York	80	[49]
37	Singapore Chinese	71	[9]	74	Kimberley	82	[49]

**Table 2:** HLA-A, -B, -DRB1 and -DQB1 Allele Frequencies in the Studied Mayos Population

Alleles	%	Alleles	%	Alleles	%
HLA-A*		39	16.7	1310	0.8
0106	0.8	40	12.5	1347	3.3
02	44.2	44	0.8	1402	0.8
03	1.7	45	1.7	1403	0.8
11	1.7	48	5.0	1406	11.8
23	2.5	51	16.7	1427	0.8
24	35.8	52	0.8	1429	0.8
26	2.5	53	0.8	1446	0.8
31	2.5				
32	0.8	HLA-DRB1*		HLA-DQB1*	
68	7.5	0101	0.8	02	2.5
		0301	1.7	0301	18.4
HLA-B*		0403	5.8	0302	62.6
7	1.7	0404	7.5	0305	0.8
8	2.5	0407	50.1	0402	10.8
14	4.2	0410	0.8	0501	0.8
15	5.8	0701	0.8	0603	3.3
18	2.5	0801	0.8	0609	0.8
27	3.3	0802	9.3		
35	25.0	1301	2.5		

**Table 3:** Genetic Distances (DA) between Mayos and other Populations (x 100) Obtained by Using HLA-DRB1 Allele Frequencies

Population	DA	Population	DA	Population	DA
Kogi	11.20	Athabaskan	63.46	Algerians	78.77
Teenek	11.38	Tuvinians	65.05	Chukchi	78.96
Mayans	13.17	Russians	65.59	Moroccan Jews	80.02
Arsario	18.13	French	66.09	Ashkenazi Jews	80.61
Mazatecan	20.44	Lebanese-KZ	68.86	Udegeys	80.69
Mixteco	23.20	Khalk-Mongolian	69.24	Eskimos	80.88
Eastern-Toba	23.32	Germans	69.39	Western Samoa	83.37
Arhuaco	24.22	Ainu	69.96	Fidji	84.19
Seri	26.73	Japanese	69.96	Kets	84.27
Nahuas	27.33	Koreans	70.74	Sardinians	84.48
Zapotecans	29.37	Spaniards	70.95	Manchu	84.87
Lakota Sioux	30.05	Danish	71.23	Evenks	86.05
Wayu	30.45	Guarani	71.62	Singapore-Chinese	86.72
Toba-Pilaga	30.72	Italians	72.40	New Caledonia	90.12
Mixe	30.98	Tlingit	72.73	Buyi	90.25
Quechuas	31.47	Moroccans	73.03	Nivkhs	90.63
Mexican Mestizos	33.93	Khoton-Mongolian	74.60	Rabaul	91.81
Cayapa	37.40	Lebanese-NS	75.36	Cape-York	95.58
Tarahumaras	37.96	Cretans	75.58	Central-Desert	95.74
Lamas	40.49	Berber-Souss	75.59	Madang	97.04
Mataco-Wichi	41.29	Macedonians	77.33	Yuendumu	98.45
Jaidukama	41.50	Chuvashians	77.66	Papua New Guinea	99.24
Aymara	42.45	Non Ashkenazi Jews	77.77	Kimberley	100
Terena Indians	43.08	Spanish Basques	78.15		
Xavantes	52.25	Koryaks	78.34		

**Table 4:** The Ten Most Frequent HLA-A, -B, -DRB1 and -DQB1 Extended Haplotypes in the Mayos Population. HF: Haplotype Frequency

Haplotypes	HF (%)	Possible Origin
HLA-A*24-B*39-DRB1*0407-DQB1*0302^a^	8.2	Amerindian
HLA-A*02-B*35-DRB1*0407-DQB1*0302^b^	7.3	Amerindian
HLA-A*24-B*35-DRB1*0407-DQB1*0302^c^	6.0	Amerindian
HLA-A*02-B*35-DRB1*1406-DQB1*0301^d^	4.2	New
HLA-A*02-B*40-DRB1*0407-DQB1*0302^e^	4.2	Amerindian
HLA-A*02-B*48-DRB1*0404-DQB1*0302^f^	3.3	New
HLA-A*24-B*51-DRB1*0407-DQB1*0302^g^	3.3	New
HLA-A*02-B*39-DRB1*0407-DQB1*0302^h^	2.7	Amerindian
HLA-A*02-B*08-DRB1*0407-DQB1*0302^i^	2.5	New
HLA-A*02-B*35-DRB1*0802-DQB1*0402^j^	2.5	Amerindian

**Table 4 footnote:**

a Only found in Mazatecans (3.3%).

b Found in Seri (18.2%); Teeneks (15.5%); Mayans (10.6%); Mixtecos (3%); Mazatecans (2.5%); Aymaras (1.7%); Peruvian Inidians (1.7%).

c Found in Mayans (5%); Teeneks (3.7%); Seris (2.3%); Lakota-Sioux (2.2%); Aymaras (2.1%); Quechuas (1.4%).

d,f,g,i Not found in any other ethnic groups.

e Found in Aymaras (2.3%) and Mayans (0.7%).

h Found in Mazatecans (10.8%); Mixe (9%); Mayans (4.2%); Teeneks (3.7%); and Terena Indians (2.3%).

j Found in Aymaras (10.4%); Peruvian Indians (9.6%); Mayans (8.4%); Nahuas (6.1%); Mixtecos (6%); Seri (4.5%); Zapotecans (3%)and Mixe (1.5%).

**Other haplotypes found are:** HLA-A*02-B*15-DRB1*0802-DQB1*0402 (1.7%), HLA-A*02-B*39-DRB1*0403-DQB1*0302 (1.7%), HLA-A*02-B*39-DRB1*1406-DQB1*0402 (1.7%), HLA-A*02-B*48-DRB1*0407-DQB1*0302 (1.7%), HLA-A*02-B*51-DRB1*1301-DQB1*0603 (1.7%), HLA-A*03-B*51-DRB1*0407-DQB1*0302 (1.7%), HLA-A*24-B*07-DRB1*0802-DQB1*0402 (1.7%), HLA-A*24-B*15-DRB1*0407-DQB1*0302 (1.7%), HLA-A*24-B*40-DRB1*0407-DQB1*0302 (1.7%), HLA-A*24-B*40-DRB1*0802-DQB1*0402 (1.7%), HLA-A*24-B*51-DRB1*1406-DQB1*0301 (1.7%), HLA-A*68-B*40-DRB1*0407-DQB1*0302 (1.7%).

**References:** [4, 17, 21, 22, 26, 30, 31, 50].

## ABBREVIATIONS

HLA: = Human leukocyte antigen
NJ: = Neighbor-joining
